# Economic burden of asthma: a systematic review

**DOI:** 10.1186/1471-2466-9-24

**Published:** 2009-05-19

**Authors:** Katayoun Bahadori, Mary M Doyle-Waters, Carlo Marra, Larry Lynd, Kadria Alasaly, John Swiston, J Mark FitzGerald

**Affiliations:** 1 Centre for Clinical Epidemiology & Evaluation (C2E2), UBC, Vancouver, BC, Canada; 2 Faculty of Pharmaceutical Sciences, UBC, Vancouver, BC, Canada; 3 British Columbia Centre for Disease Control (BCCDC), Vancouver, BC, Canada; 4 Department of Medicine, Respiratory Division, UBC, Vancouver, BC, Canada

## Abstract

**Background:**

Asthma is associated with enormous healthcare expenditures that include both direct and indirect costs. It is also associated with the loss of future potential earnings related to both morbidity and mortality. The objective of the study is to determine the burden of disease costs associated with asthma.

**Methods:**

We performed a systematic search of MEDLINE, EMBASE, CINAHL, CDSR, OHE-HEED, and Web of Science Databases between 1966 and 2008.

**Results:**

Sixty-eight studies met the inclusion criteria. Hospitalization and medications were found to be the most important cost driver of direct costs. Work and school loss accounted for the greatest percentage of indirect costs. The cost of asthma was correlated with comorbidities, age, and disease severity.

**Conclusion:**

Despite the availability of effective preventive therapy, costs associated with asthma are increasing. Strategies including education of patients and physicians, and regular follow-up are required to reduce the economic burden of asthma.

## Background

**Figure 1:**
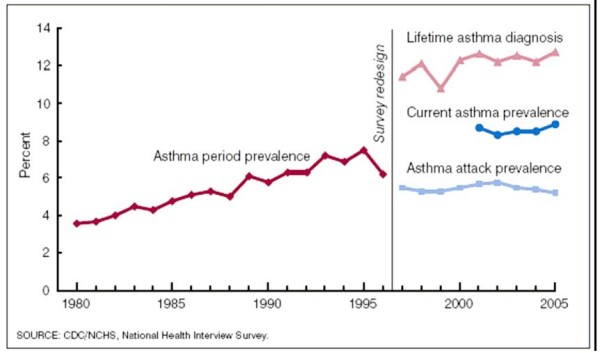
**Trend in prevalence of asthma**.

Asthma is an inflammatory disorder of the lungs that affects people of all ages and is a significant source of morbidity and mortality worldwide [1,2]. Approximately 300 million people in the world currently have asthma and recent decades have shown a concerning increase in the prevalence of this condition in both children and adults (Figure 1). There has been concerning increase in the prevalence asthma in both children and adults) [3]. If the current trends continue, it is estimated that there may be an additional 100 million more asthmatics by 2025 [4].

The economic costs associated with asthma are estimated to rank as one of the highest among chronic diseases due to the significant healthcare utilization associated with this condition. Numerous studies have been published evaluating the economic burden of asthma on society and individuals. However, a systematic review of the financial impact of asthma has not yet been performed [5-8]. The goal of this systematic review is to evaluate and synthesize the current literature regarding the economic burden of asthma. The evaluation of the cost of asthma from both a social and economic perspective is necessary for an optimal allocation of resources as well as the betterment of patient care. This study sought to address the following question: "What are the direct, indirect (productivity), and overall costs associated with asthma?"

## Methods

### Literature Search

A systematic review was conducted to identify English language articles published between 1966 and January 2008 in which the costs of asthma were included. Only studies reported in English (as there was no translator available) and published literature, were included.

The following electronic databases were searched using MEDLINE, EMBASE, Cumulative Index to Nursing and Allied Health Literature (CINAHL), Cochrane Database of Systematic Reviews (CDSR), Health Economic Evaluation Database (OHE-HEED), and Web of Science. Search terms were investigated, including: "asthma", "direct service costs", "cost of illness", "cost- benefit analysis" and "health care costs". Duplicate citations were identified and removed using RefWorks online bibliographic management tool.

### Study Selection

The titles and abstracts of all publications identified through the primary literature search were independently reviewed by two investigators. The inclusion and exclusion criteria used for study selection are outlined in Table 1. The total of four non-English abstracts were retrieved and excluded from the literature search. The full text of all potentially eligible papers determined after the first level of screening was reviewed to ensure that each paper met the inclusion criteria for population and outcomes of interest.

**Table 1:** Inclusion and Exclusion Criteria

**Inclusion Criteria**	**Exclusion Criteria**
• English language	• Conference abstracts, case reports, letters, comments, editorials and review papers
• Studies that consider the costs of asthma from either the individuals', the health services', and/or society's perspective	• Studies that consider asthma with other comorbidities (such as allergies, COPD, etc)
	• Pharmacodynamic or pharmacokinetic studies
	• Studies that did not quote costs in the results section
	• Animal or in vitro studies

The types of cost related to illness were divided into direct and indirect costs and were defined as follows: direct costs related to direct health service costs and included alternative treatment/medications, physiotherapy/chiropractic, peak flow meters, primary care consultations, paid help for housekeeping, hospital emergency and outpatient attendance, ambulance and other transportation, and hospital admissions. Indirect costs were those applicable to individual patients, their families, and lost opportunities for work or education. The total cost is an aggregate of both direct and indirect costs. For cost data from the United States (US), costs were converted to 2008 US dollars using the medical care component of the consumer price index from the US Bureau of Labor Statistics.

For cost data from non-US countries, figures were first converted to 2008 currency values using that country's consumer price index. Figures were then converted to 2008 US dollars, using currency exchange rates of Australian dollar (AUD)$1.00 = United States dollar (USD)$0.637, Canadian dollar (CAD) $1.00 = USD$0.806, (Euro currency code) EUR€1.00 = USD$1.281, and Great Britain pound (GBP)£1.00 = USD$1.427. If the year of the cost data was not reported, it was assumed to be the publication year of the article. In all cases, both the original cost figures provided in the publications as well as the equivalent costs in 2008 US dollars were reported.

### Data Abstraction

A standardized data abstraction form was used for all publications included in this study. The data abstracted included the following information: manuscript authors, year of publication, study design and duration, patient characteristics (population, age and gender), method of cost calculation, direct costs, productivity (indirect) costs, and total costs. Discrepancies in data abstraction were resolved by consensus.

### Methodological Quality Assessment

The quality of the economic studies was assessed using a customized version of the Drummond and Jefferson criteria (Table 2) [9]. Quality criteria were scored as positive, negative, or unclear. Study quality was assessed by one reviewer and confirmed by a second reviewer. Economic evaluations that scored 50% or more of the items positive were defined as studies of high methodological quality, whereas less than 50% was considered low methodological quality. The 50% score was arbitrarily chosen as a mean cutoff to create binary categories.

**Table 2:** Criteria for evaluating an economic analysis based on Drummond and Jefferson assessment method*

1. Was a well-defined question asked in an answerable form?
2. Was a comprehensive description of the competing alternatives provided?
3. Was there evidence that the program's effectiveness was established?
4. Were all the important and relevant costs & consequences identified?
5. Were costs and consequences measured accurately with appropriate physical units?
6. Were costs and consequences credibly valued?
7. Were costs and consequences adjusted for differential timing?
8. Was an incremental analysis of costs and consequences of alternatives performed?
9. Was a sensitivity analysis performed?
10. Did the presentation and discussion of the study results include all issues of concern to users?

*Note: All items have three possible responses, Yes (+), Cannot tell (N/A) and No (-).

### Statistical Analysis

Due to the heterogeneity in cost analysis and reporting across the studies, a quantitative meta-analysis to aggregate cost data could not be performed. Specifically, the resources used to derive direct healthcare and productivity costs varied substantially between the studies, as did the type of currency (USD, GBP, CAD, etc). In the absence of the ability to complete a meta-analysis, we opted to complete a qualitative analysis. There were not sufficient homogeneity in terms of participants, interventions, and the way outcomes were defined and measured, to provide a meaningful summary for considering the meta-analysis.

## Results

### Literature Search

**Figure 2:**
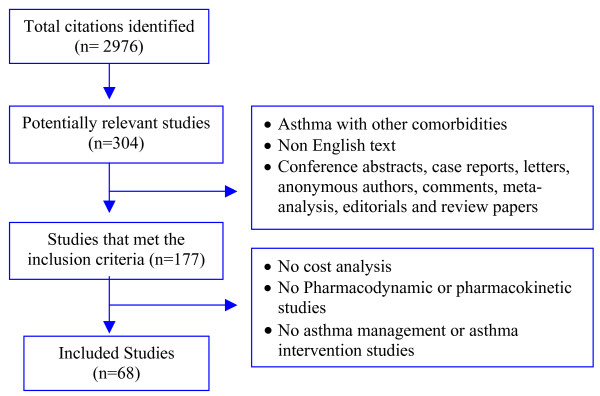
**Results of systematic literature search**.

The primary literature search identified 2,976 citations. After removing duplicate citations we were left with 2,073 unique citations for screening. The manual screening of all 2,073 titles and abstracts yielded 307 articles that contained primary clinical data evaluating the cost of asthma. Of the 307 full articles retrieved and reviewed by the investigators, 68 met the inclusion criteria (Figure 2).

### Quality Assessment

Most of the studies clearly described the inclusion and exclusion criteria and the population as well as specifying the primary outcome measures. The study designs were cohort (n = 43), cross- sectional studies (n = 22), and case-control studies (n = 3). Of the 68 studies, only six completed sensitivity analysis [10-15]. Common perspectives of an economic evaluation were the societal perspective (all costs and outcomes experienced by all those who are significantly affected by the intervention) and the healthcare perspective (only health costs and outcomes). Thirty-two studies mentioned the perspective (all societal) of the economic evaluation [10-43]. Twenty-six studies calculated both direct and indirect costs, which suggests, although not explicitly mentioned, that these studies also adopted a societal perspective. Ten studies only calculated direct medical costs and thus, adopted a healthcare perspective. Three studies calculated an incremental cost effectiveness ratio (ICER) [10,19,21]. The uncertainty of the outcomes was presented through sensitivity analyses in six studies [10-15]. The mean score of the quality assessment regarding the economic evaluation was 6.1 out of a maximum of 10 (Standard Deviation (SD) 1.43; range, 3 to 9).

### Characteristics of the Selected Studies

Of the 68 studies identified in the literature review, twenty-three used data derived from the US, twenty-five from European countries, eight from East Asia and the Pacific regions, five from Canada, and seven were from other countries. Twenty-eight studies reported mean or median per patient direct and indirect costs of asthma, thirteen only reported the total direct costs of asthma, and fifteen studies reported annual or total asthma related healthcare expenditures. The economic impact in five studies was based on charge data (not costs) [25,44-47].

The studies presented in this review used several methods of cost calculation. The most common method was to abstract mean patient resource utilization, such as the number of clinic visits, hospitalizations and procedures, through chart reviews or insurance databases, and to combine these data with the mean unit costs of each resource, derived from local or national accounting databases. An alternative method was to follow a cohort of subjects via a cost-accounting system to capture the charges or costs incurred over time in the management of asthma symptoms.

### Direct Versus Indirect Costs of Asthma

Cost of asthma includes the components of both direct and indirect costs. Direct costs include inpatient care, emergency visits, physician visits, nursing services, ambulance use, drugs and devices, blood and diagnostic tests, research, and education. Indirect costs or morbidity costs include school days lost, traveling, waiting time, and lost productivity for the caretaker of asthmatic children. Direct costs have been shown to exceed indirect costs, and the major components of the direct medical costs found were pharmacological expenditures and hospital admissions. An increase in direct medical costs can potentially lead to a reduced total cost of care if it results in a disproportionately greater reduction in indirect costs due to improved clinical outcomes. In our systematic review, nine studies have found that the direct cost of asthma accounted for the greatest part (53–100%) of the overall cost [11,14,18,29,42,47-50] (Table 3). Of these, five were considered high quality studies. However, some of the reviewed cost-of-illness studies estimated the healthcare costs associated with asthma, but they took the broader societal perspective and also included the impact of morbidity and mortality on employment, productivity and other social costs. In five studies, the indirect costs greatly exceeded the direct costs [13,17,22,51,52]. The quality of all but one of the above studies was also high [51] and indirect costs accounted for 52–75% of the overall costs (Table 4).

**Table 3:** Studies were direct cost exceeded indirect cost of asthma

**References No**.	**Country**	**Study duration**	**Sample size**	**Total direct cost/person**	**Mean annual direct cost/person**	**%**	**Total indirect cost**	**Total cost**	**Mean annual cost/person**
**Institutional studies**									

**11**	US	1 yr	401	-	3,307	65	1,801	-	4,158
**18**	Canada	1 yr	149	196,898ϕ	-	74	67,729	264,627	-
					-	-	-	-	-
**42**	Canada	1 yr	339	-	1,200 x	88	157	-	1,357
					752 y	100	-		752
					85 z	56	66		151

**Regional studies**									

**47**	Australia	1 yr	1.2 m	273	-	77	81	354	-

**National studies**									

**14**	US	1 yr	4.7 m	7,301	-	88	955	8,256	-
**29**	Canada	1 yr	N/A *	397	-	61	257	654	-
**48**	Switzerland	1 yr	N/A *	860	-	61	553	1,413	-
**49**	US	1 yr	N/A *	3,822	-	53	3,367	7,189	-
**58**	US	10 yrs	14.2 m	8,665	-	57	6,583	15,248	-

All costs are converted and adjusted into 2008 US dollars

Cost in regional and national studies are in million of dollars

Φ: of total study sample size, x = societal perspective, y = ministry of health perspective, z = patient perspective

m: million; yr: year; N/A*: data not available

**Table 4:** Studies were indirect cost exceeded direct cost of asthma

**References No**.	**Country**	**Study duration**	**Sample size**	**Total indirect cost**	**Mean annual indirect cost/person**	**%**	**Total direct cost**	**Total cost**	**Mean annual cost per person**
**Institutional studies**									

**13**	Spain	1 yr	333	-	2,749	69	1,221	-	3,970
**17**	Italy	1 yr	500	-	1,068	52	970	-	2,038
22*	US	1 yr	638	-	n/a	55	488	-	727
**50**	Denmark	1 yr	115	822,067	-	67	402,668	1,224,735	-

**National studies**									

**51**	Germany	1 yr	52,794	n/a	-	75	-	4.43 b	-

All costs are converted and adjusted into 2008 US dollars

* n/a: data not available; b: billion; yr: year

### Direct Cost of Asthma

#### Hospital Admission

In this systematic review, the largest amount of direct costs found were those allocated to in-patient hospitalization, accounting for 52 to 86% of the overall asthma-related costs in seven studies, [28,30,38,42,53-55] and 47 to 67% of total direct costs in another five studies [14,15,20,48,49] (Table 5).

**Table 5:** Studies that represented inpatient care cost as the largest proportion of total direct cost or total asthma related cost

**References No**.	**Country**	**Study duration**	**Sample size**	**Cost of inpatient care/person**				**Total direct cost**	**Total asthma related cost/person**	**Total asthma related cost**
				**Mean**	**SD**	**95%CI**	**%**			

**Institutional studies**										

**15**	Canada	1 yr	940	614^¥^	-	-	-	1,421	-	-
**28**	Australia	1 yr	245	397	-	-	-	-	779	-
**30**	France	1 yr	17 **	229	282.4	-	52	-	-	6,856
**38**	UK	1 yr	29*	439	816	0–2,895	57	-	-	773
**42**	Canada	1 yr	339	591	-	-	77^ψ^	-	766	-
**48**	Switzerland	1 yr	589	928	-	-	47^ξ^	1,779	-	-
**52**	Spain	3 mo	126	1,932	-	1,529–2,353	83	2,338	-	-
**53**	France	1 yr	94**	64,675ϕ	-	-	86	-	-	745,847

**Regional studies**										

**54**	US	1 yr	530,000	7.8 m	-	-	-	-	-	20 m

**National studies**										

**14**	US	1 yr	4.7 m	4 b	-	2 – 6 b	54	7 b	-	-
**20**	Singapore	1 yr	27,164**	12 m	-	-	-	25 m	346	49 m
**49**	US	5 yrs	463,500**	1.6 b	-	-	67	4 b	-	7.2 b

All costs are converted and adjusted into 2008 US dollars

¥ from the societal perspective; * Number of the trial families admitted to hospital, out of 94 trials; **average number of hospitalization for asthma in one year; ξ: Hospital care found to be a major cost factor only in the adult group of the study;

ψ: From ministry of health (MOH) perspective. It accounted 43% of total cost from a societal perspective;

ϕ:of total study sample size; m: million; b: billion; mo: month; yr: year

There are several factors that can contribute to higher total hospital costs. Two studies found the following variables correlated significantly with higher hospital costs: older patients, significant comorbidities, intensive care unit (ICU) admission, increasing severity, and prolonged length of stay. In both studies, a small proportion of asthmatics accounted for a large proportion of the total hospital costs [36,56]. A similar result has also been observed in another study, possibly because of poorly controlled patients with more severe asthma [14].

Stanford and associates reported resources such as nursing care, respiratory therapy, and ED-specific supplies along with equipment use and physician fees to account for the majority of hospital costs of asthma; 44%, 11% and 53% of the costs, respectively [36]. The results of the cost estimation for asthma hospitalization in Quebec for the year 1994/95 indicated that of the total cost of $23.3 million, the greatest proportion was accounted for by pediatric patients ($11 million) [57]. Similar findings in a retrospective cohort study also suggested that although teaching hospitals in their study were not found to have higher charges, children's hospitals appeared to be more expensive due to the inherent responsibilities of a teaching hospital, but also due to the fact that they act as regional referral centers with specialized services for children [25].

A patient's characteristics can also affect the cost of hospitalization. In a study conducted in France, the cost of a hospital stay was compared between a well-managed asthmatic group and a poorly managed one before hospitalization based upon guidelines (11 criteria judged by experts). The poorly managed group was older, tended to include more smokers, spent half as much time in ambulatory care, and had a shorter length of hospitalization. The cost of a hospital stay was found to be 1.72 times higher in the better managed group, due mainly to the differences in the length of the hospital stay. However, in the unmanaged group, non conformity to the treatment for the attack resulted in a cost excess ($4,900 vs. $4,065 p < 0.05) [58].

Over a ten-year period in United States, hospital inpatient care represented the largest component cost of direct medical expenditures in 1985. However, in 1994, medications were reported to be the largest component cost of direct medical expenditures. The annual estimates increased from approximately $1.4 billion (1985 adjusted dollars) to $2.5 billion. This increase was due to an estimated 103.2% increase in total number of prescribed medications and an estimated 169.3% increase in average unit cost per medication [50].

#### Asthma Cost and Hospital Characteristics

Our review showed that there are associations between asthma-related costs and the stratification of hospitals by geographic region, ownership, location, and teaching status. In this systematic review, five studies explored the relationship between hospital "characteristics" and the cost of asthma. A large, comprehensive administrative database examined the association of selected hospital characteristics with cost among pediatric patients with asthma and its severity by hospital type in the state of New York. Hospital types were classified into teaching versus non-teaching and private versus public. They reported the mean cost of asthma to be higher in private hospitals, ($1,868 vs. $1,771) and non-teaching hospitals ($1,876 vs. $1,528). Although after adjustments for patient and hospital covariates, the differences in mean cost between public and private hospitals did not remain significant [59]. In contrast, a high-quality study conducted at a teaching hospital in an urban setting in Canada, found total operating expenses per in-hospital patient day to be $681.70 for teaching hospitals and $496.81 for non-teaching hospitals, for the fiscal year 1990 to 1991 [18]. However, in another cohort study, after adjusting for patient and hospital characteristics, they found no differences in hospital charges between teaching and non-teaching hospitals [25].

In a cross-sectional study, the mean total charges, after adjusting for significant covariates including severity of illness, income, and payer, were found to be significantly higher at investor owned ($4,203) as opposed to nonprofit ($3,640) or public hospitals ($3,620). Furthermore, the average charges were found to be higher at urban teaching ($4,230) and lower at rural institutions ($2,910) compared with urban non-teaching hospitals ($3,424) [44]. However, a five year-population-based study yielded contradictory findings regarding costs per hospital discharge. In this study, public hospitals reported the highest costs per discharge when compared with not-for-profit and for-profit hospitals ($656.84 vs. $514.08 and $411.95, respectively). They included the asthma patients discharged with referral to an outpatient unit, who generally had higher costs than those patients discharged who had made a full recovery [32].

#### Asthma Medications

Medications were found to be another major contributor to the cost of asthma. Eighteen studies reported "medications" as forming the largest proportion of the direct costs related to asthma, accounting for 38%–89% of the total cost [11,13,15,16,19,22,23,27-29,35,37,43,46,47,52,56,60] (Table 6).

**Table 6:** Studies that represented medication costs as the largest component of total asthma related cost

**References No**.	**Country**	**Study duration**	**Sample size**	**Cost of medication/person**				**Total direct cost**	**Total cost**	**Mean annual cost/person**
				**Mean**	**SD**	**95%CI**	**%**			

**Institutional studies**										

**11**	US	1 yr	401	2,070			50	4,101		
**13**	Spain	1 yr	333	552	454		45	1,221		3,971
**15**	Canada	1 yr	940	56¥				82		
**16**	Italy	1 yr	446	240		181–299	47 ν			1,186
**19**	Turkey	1 yr	118	1,388	109		81	1,713		
**22**	US	1 yr	638	141			53 ψ	488	727	
**23**	France	1 yr	234	-			60–75 ξ	-		
**27**	Sweden	1 yr	220	463			56	822	0.62 b	2,655
**28**	Australia	1 yr	245	55			89^¢^			235 ¥
**35**	Switzerland	1 yr	263	814	735		70	1,156		
**55**	Thailand	1 yr	183	72	111		47	154 §		
**60**	Estonia	1 yr	1,423	154,140ϕ			53 ψ	3.60 m		203

**Regional studies**										

**37**	US	1 yr	25,614	-			38			679
**47**	Australia	1 yr	1.2 m	147 m			54	273 m	354 m	

**National studies**										

**29**	Canada	1 yr	N/A*	164 m			-	404 m		
**43**	US	1 yr	2.52 m	495 m	66		43		2.3 b	
**51**	Germany	1 yr	52,794	0.58 b			84	0.69 b	2.74 b	

All costs are converted and adjusted into 2008 US dollars

Cost information of study 46 was not available

¥: From the patient's perspective; ¥: Total annual cost per person of asthma management to *individuals*; ν: 47% of mean annual direct cost per patient;

^¢ ^:The major component of the individual cost; N/A*: All asthmatic patients in 1990; ξ: 75% of stage 2&3 and 60% of stages 1&4 (the total amount is not available); §: hospital resource utilization; ψ: 53% of total medical expenditure; ϕ: of total study sample size; m: million, b: billion; yr: year

In a large-scale study, the costs of asthma medication were found to be the largest cost factor in children ($382.09 or 41.3% of total direct cost) whilst it was reported as being lower in adults. Furthermore, hospital care was found to be a major cost factor in adult patients ($928.28, accounted for 46.5% of total direct cost) [48]. Besides age, severity of disease was found to be another factor affecting the cost of medications. In a high quality study in Switzerland between 1996 and 1997, the cost categories differed greatly between those with and without exacerbations. In the latter, medication costs represented 70% while hospitalization costs were 10% of total cost. However, in those with exacerbations, medication costs contributed only 28%, but hospitalization costs contributed 63% of total costs [35]. Moreover, differences in the perspectives of patients, society, and the Ministry of Health could affect both their behaviors and the asthma related costs. In two different high quality studies, the cost of asthma in Canada was estimated from the perspectives of society, the Ministry of Health, and the patient. Hospital admissions were reported to be the highest component of total cost in adults from a societal perspective in the one study [15], and the highest component of total cost in children from both societal and the Ministry of Health's perspectives in the second study [42]. However, medication costs were found to be the largest single component of direct costs from the patient's perspective, in both studies [15,42].

Besides hospitalization and medications, some patients were shown to have much higher or lower costs, depending on the actual utilization of medical services.

#### Physician Visits/Outpatient Clinic

Outpatient services provided by hospitals may be in the form of clinics, or be more similar in practice to a group medical practice within a hospital. These outpatient visits provide the same kind of care that is provided in a physician's office; it is thus anticipated that the costs will be the same. In a recent retrospective study, the largest proportion of direct costs was due to outpatient clinic costs (48.5%) and the majority (~76%) of this cost was due to completing pulmonary function tests (44%) and skin-prick tests (34%) [61]. Similarly, two other studies reported "physician visits or office-based visits" to be the largest expense of asthma related direct costs accounting for 55–58% of direct costs [21,39]. Likewise, a cost-of-illness study in United States over the 10-year period from 1985–1994 reported an increase in total estimated annual asthma-related physician office visits combined with an increase in the average charge per visit, which accounted for an 82% relative increase in office visit expenditures [50].

#### Cost of Asthma and Category of Service

Unscheduled consultations found to be major components of primary care costs associated with the management of asthma. Prescribed medication for maintenance treatment was also found to contribute significantly to the total asthma related costs. However, the results from studies comparing these two services have given conflicting results. In a large high-quality cohort study in eight countries from the Asia-Pacific region, direct costs of asthma were estimated as: total costs, urgent (unscheduled) versus maintenance costs, and drug versus non-drug costs. The study reported that the urgent care costs were higher than maintenance costs in the participating areas: Singapore, Hong Kong, Malaysia and China, representing 62% of the total costs [31]. In contrast costs of maintenance therapy were reported to be more important in adults and children, respectively accounting for 67% of total health services in one study [62] and 55% and 73% of the total direct costs of treatment in another study [63]. None of the two latter studies were of high quality and thus, may not be a true reflection of the overall economic impact of asthma.

Another cohort study classified the encounters into four categories of services: non-urgent outpatient visit, urgent care visit, pharmacy refills, and hospitalization. This study showed that two-thirds of the asthma care costs were attributable to non-urgent outpatient care and prescriptions; only one third were found to be attributable to urgent care and hospitalizations. This was possibly due to better access of enrollees to preventive asthma care, resulting in fewer acute exacerbations requiring emergency department (ED) visits or hospitalizations [33]. Similarly, a recent study conducted in Taiwan found that almost three-fourths of asthma related costs was attributable to office and outpatient care; and only one-fourth were attributable to urgent care and hospitalization. The authors felt their findings to be reasonable given the regular clinical and follow up these patients received, which diminished the need for urgent care and hospitalization [40].

#### Insurance Coverage

In this systematic review, we found that adequate insurance coverage in patients with asthma correlated with a reduced use of urgent or emergent care. Two studies investigated the influence of insurance coverage on medical resource utilization and costs. In the first, which was a high-quality cohort study, the patients who were insured reported for every visit that they used more primary care services (at higher costs) and less emergency department services (at lower costs) than patients who were uninsured. However, patients who were uninsured on some visits were reported to have higher primary care, emergency department, and inpatient costs than patients in any other insurance category, suggesting a greater severity of illness among these patients. An additional reason is that they also lacked insurance coverage to purchase more appropriate controller asthma medications [39]. Similarly, in the second study, patients with supplementary insurance coverage reported a lower overall resource utilization rate. Total costs amounted to $2,446 for patients without supplementary insurance coverage and $2,092 for patients with such coverage [64].

### Indirect Cost of Asthma

Economic assessments of asthma included in the reviewed studies indicated that decreased productivity at work and school represent a considerable proportion of the disease burden, specifically adding to indirect health costs. To accurately assess health-related work impairment, it is important to take into account both time lost from work, or absenteeism, as well as loss of productivity while at work. Productivity losses were measured as the days lost from productive activities because of asthma, the days that patients worked despite asthma symptoms (restricted days), and travel and waiting time associated with receiving outpatient asthma care. Twelve studies included loss of productivity due to absenteeism from work/school as the largest single indirect cost [11,13-16,20,27,42,43,48,51,65], and loss of school/work days was found to be the largest category of indirect cost in eight studies [10,18,21-23,26,49,50] (Table 7). The indirect cost of asthma was not evaluated in eleven studies [19,30,34,35,39,54,62,64,66-68]. The quality of the eleven studies, except for two of them, was all estimated to be above average.

Children with asthma have much higher indirect costs than the average child as the costs of parents missing work due to the child's asthma is also an indirect cost. In a study conducted in Hungary, high consumption of indirect resources was observed for both the adult and pediatric population. However, the indirect costs represented a much higher proportion of total costs for pediatric patients compared with adult patients (52% vs. 21%). Furthermore, there was a statistically significant increase in the cost of lost work between parents of patients with good control and parents of patients with poor control [26].

**Table 7:** Studies that represented loss of productivity due to absenteeism from work/school as the largest proportion of total indirect costs

**References No**.	**Country**	**Study duration**	**Sample size**	**Productivity loss/person**				**Total indirect cost**	**Total asthma related cost**
				**Mean**	**SD**	**95%CI**	**%**		

**Institutional studies**									

**10**	US	1 yr	3528	83	-	-	-	148	-
**11**	US	1 yr	401	1,370	-	-	61	2,234	-
**13**	Spain	1 yr	333	553,569 ϕ	-	-	-	915,674	-
**15¥**	Canada	1 yr	940	935	-	-	92	1,018	-
				1,411	-	-	50	2,836	-
**16**	Italy	1 yr	527	416	-	261–573	53	663	-
**18**	Canada	1 yr	149	44,623	-	-	-	67,729	-
**21**	US	1 yr	443	1,437	3732	-	-	1,788	-
23*	France	1 yr	318	-	-	-	-		-
**26**	Hungary	1 yr	378 x	540	-	-	-	1,132	-
			711 y	170	-	-	-	810	-
**27**	Sweden	1 yr	115	1,399	-	-	76	1,778	-
**42**	Canada	1 yr	339	66	-	-	44	150	-
**50**	Denmark	1 yr	115	760,853 ϕ	-	-	62	822,067	1.2 m

**National studies**									

**14**	US	1 yr	35000	315 m	-	113–517	33	955 m	8,256 m
**20**	Singapore	1 yr	142,300	18.5 m	-	-	37	24.31 m	49.36 m
**43**	US	1 yr	2.52 b	821 m	-	-	-	1,124 m	2.3 b
**48**	Switzerland	1 yr	N/A	414 m	-	-	75	551 m	1,413 m
**49**	US	1 yr	N/A	1,448 m	-	-	-	4.2 b	7.2 b
**58**	US	10 yrs	14.2 m	1,902 m	-	-	-	6,583 m	15,248 m

All costs are converted and adjusted into 2008 US dollars

* Cost information of studies 65 and 22 were not available

¥: 92% from the patient perspective and 50% from the societal perspective; ϕ: of total study sample size; N/A: Number of asthmatics patients in US in 1985

x: adult and pediatric patients, y: pediatrics; m: million; b: billion

### Cost of Asthma and the Associated Risk Factors

#### Severity

In our systematic review, disease of greater severity, was associated with a higher total costs. Twenty-two studies suggested more severe disease to be a major factor influencing the increase in asthma- related costs [11,13,15,17,19,21-23,27,30,31,35,36,41,46,51,53,54,58,62,66,67] (Table 8). In two studies, the severity of illness was classified into mild, moderate and severe categories. The first study, conducted in Spain, reported that the per-patient cost ratio of asthma increased to 1 to 1.5 to 2.6 for the three levels of asthma. The second study, from US, reported that the per-patient cost ratios increased to 1 to 1.4 to 2.4 for pediatric patients and 1 to 1.5 to 2.9 for adult asthma patients having good, moderate, and poor asthma control, respectively [13,26]. Similarly in a study in the US over a two year period, asthma costs per patient were found to be five times higher for those whose asthma was categorized as severe than for those with mild asthma ($1,579 vs. $298) on a per patient basis [67]. Another Spanish study revealed that the cost of a moderate asthma exacerbation was 4-fold greater than that of a mild exacerbation, and the cost of a severe exacerbation as much as 12 times that of a mild exacerbation. The cost analysis, according to prior asthma severity, also indicated that the cost attributed to a patient with severe persistent asthma was 2.2 times higher than that of an exacerbation in a patient with intermittent asthma. This indicates that severe exacerbations were found to be more harmful to the patient and much more costly to the health system regardless of the prior disease severity [53]. In one high quality study in the US, dividing patients with persistent asthma into mild, moderate, and severe categories showed that the average annual direct cost of a patient with severe, persistent asthma to be 1.3 times the cost of a patient with moderate, persistent asthma and 1.7 times the cost of a patient with mild, persistent asthma [21]. Moreover, another high-quality study completed in Spain between 1994 and 1995 reported the costs of asthma for patients with severe disease to be almost three-times higher than for those with moderate asthma and five-times higher for those with mild illness [13]. In 1999, a retrospective cohort analysis of a representative data set of 12,203 patients with asthma in the United Kingdom (UK) made a comparison of healthcare costs between patients who had an asthma attack and those who did not. Average total costs per patient was reported to be 3.53 times higher in the group who reported asthma attacks than in the non-attack group, indicating that the cost of managing patients who experienced an acute asthma attack impinged heavily on healthcare budgets [69]. Similarly, in a large-scale Swiss study, total costs in patients with attacks were 2.38 more compared with patients without attacks [48].

**Table 8:** Studies that found influence of severity on asthma- related costs

				**Severity**												
					
				**Intermittent**			**Mild**			**Moderate**			**Severe**			**Subgroups**
					
**References No**.	**Country**	**Study duration**	**Total n**	**n**		**Cost**		**n**		**Cost**	**n**	**Cost**	**n**		**cost**	
**Institutional**																

**11**	US	1 yr	401	-		-		200		3339	137	5,716	64		16,168	
**13**	Spain	1 yr	333	-		-		140		257,979	116	385,239	77		679,091	
**15**	Canada	1 yr	940	-		-				1,799		2,466			4,344	Societal perspective
						-		256		744	459	862	225		1,192	Ministry perspective
						-				877		1,079			1,763	patient perspective
**19**	Turkey	1 yr	118	4		202		54		1,006	46	1,954	14		4,081	
**21**	US	1 yr	3002	-		-		787		5,390	1194	6,838	988		9,020	
**23**	France	1 yr	318	32		435		78		1,134	91	1,977	33		4,598	
**27**	Sweden	1 yr	115	53				359			62		4,473			
**30**	France	1 yr	261	-		-		108		606	58	704	63		561	Good control
						-				1,184		808			1,426	Moderate control
						-				329		741			4,476	Poor control
**35**	Switzerland	1 yr	422	14		511		31		1,440	42	3,487	72		5,228	
**36**	US	1 yr	3223	515		2,875		431		3,973	101	6,303	27		19,820	
**46**	Thailand	1 yr	511	-		104		-		125	-	182	-		224	
**52**	Spain	3 mo	126	-		-		-		515	-	1,926	-		5,544	
**53**	US	1 yr	1038	-		-		-		536	-	1,255	-		627	Good control
						-		354		651	200	1,119	347		1.214	Moderate control
						-				1,903		1,953			1,808	Poor control
**57**	France	1 yr	169	66				3,942			53		4,608			Group P
				23				4,527			27		9,574			Group A
**62**	Turkey	1 yr	183	-		-		124		806	36+23		1,729			
**67**	US	2 yrs	2213	-		-		1,007		463	237	374	969		2,362	
**41**	Australia	1 yr	193	No visits		One visit/yr		2 Visits/yr		>2 Visits/yr		Urgent visit		Hospitalization		
				n	$	n	$	n	$	n	$	n	$	n	$	
				35	80	26	195	38	207	55	202	31	298	8	819	

**Regional**																

**50**	Denmark	1 yr	115	16		755		47		8,856	48	13,201	4		40,677	
**17**	Italy	1 yr	500	174		1,165		134		1,692	153	2,507	39		5,382	

**National**																

**66**	11 countries	1 yr	1201	-		1,143		-		1,113	-	1,213	-		1,480	
	10 countries		1124													

All costs are converted and adjusted into 2008 US dollars

Group P: not managed; group A: managed; * Cost information of studies 31 and 22 were not available.

#### Poor Asthma Management

The mean annual cost per patient increased as the level of disease control decreased. Seven studies reported that "poor asthma control" was associated with an increase in healthcare and elevated costs [14,16,26,30,31,51,54] (Table 9). All but one were rated as a high quality study. In a study by Lai et al, the relationship between asthma control status, measured using a derived Asthma Control Test (ACT) score, and utilization of healthcare and its cost in eight Asia-Pacific areas were explored. ACT questions asked patients to report for the previous four weeks, limitations to activities; shortness of breath; nighttime awakening; use of rescue medication; and perception of control. Completion of the ACT resulted in a potential score of between 5 and 25; a score of ≥ 20 indicated "well-controlled" asthma and a score of <15 suggested "poorly controlled" asthma. The mean per-patient annual cost of asthma management for patients with a derived ACT of <15 was $861; $319 for patients with a derived score 15–19; and $193 for patients with a derived ACT score of ≥ 20. A higher derived ACT score was associated with a significantly lower annual expenditure on asthma management. This provides new evidence quantifying the link between asthma control and unscheduled healthcare resource use and cost. Poor asthma control was found to be associated with a greater likelihood for hospitalization and unscheduled physician visits in the previous year. Not surprisingly higher asthma related costs were also found [31].

**Table 9:** Studies that found increased cost of asthma associated with poorly controlled asthma

**Reference No**.	**Country**	**Study duration**	**n**	**Asthma control**		**Cost stratified by severity**		
						
						**Low**	**Moderate**	**High**
**50**	Denmark	1 yr	39	Well treated		8,948		
			76	Poorly treated		11,523		
								
								
**53**	US	1 yr	515	Well treated		536	651	1,903
			294	Intermediate		1,255	1,117	1,954
			128	Poorly treated		627	1,214	1,808
								
**14**	Italy	1 yr		Well treated			593	
			527	Poorly treated			2,099	
								
**20**	Hungary	1 yr	248	Pediatrics	Well treated		973	
			98		Intermediate		1,374	
			10		Poorly treated		2,651	
			352	Adults	Well treated		554	
			254		Intermediate		861	
			88		Poorly treated		1,632	

All costs are converted and adjusted into 2008 US dollars

Cost information of studies 30 and 31 were not available

#### Disability Status and Comorbidity Conditions

An association between asthma patients with co morbidities and higher costs was also found. Three studies reported that costs increased significantly amongst asthma patients with comorbidities/comorbid conditions [24,35,54]. In a high quality study, diseases such as hemiplegia, neurological disorders, psychosis, and acquired immune deficiency syndrome (AIDS), which were associated with significant resource use in general, were found to be highly significant cost drivers for the asthma cohort. More importantly, low-cost high prevalence diseases such as lower respiratory tract infections were also found to be significant cost drivers as well [34].

The high costs among unemployed or retired patients constituted an important finding because these patients were found to be most likely to have a poor overall prognosis in terms of morbidity and mortality. In a study conducted in Switzerland, the highest total costs were observed among jobless patients and those receiving disability payments [63]. Similarly, in another study conducted in Canada, illness-related disability was found to be the largest component of indirect costs ($76 million) [29]. In another high quality study, total costs among worker's with asthma, with disability claims were reported to be approximately three times higher than among disability claimants in the employee control sample ($14,827 vs. $5,280). Although treatment for asthma itself accounted for 16% or more of total costs, comorbid conditions accounted for an additional 13% or more. For workers with asthma, wage-replacement costs for workdays lost (40%) were found to be almost as much as medical care (43%) [10]. Similarly, in a high quality study, disability pensioners and homemakers were found to have higher costs than severely ill patients [15].

#### Age

Age was also found to have an association with the cost of asthma, but there were conflicting reports. Younger age was found to be a significant predictor of higher costs in seven studies [12,14,22,42,62,70,71]. However, seven studies reported "older age" to be significantly more likely to have higher costs [15,27,36,39,54,56,58], or costs of asthma to be increased as patients' age increased [35,45,48,52,60,66]. One study reported asthma-derived direct costs to be double in elderly compared to younger asthma patients, mainly due to higher costs of hospitalization and medication [68]. Two other cohort studies found totally different results. The first, conducted in the US, after stratifying patients into five age groups (5–9, 10–14, 15–19, 20–29, and 30–34) years, found that adolescents (15–19) years old had both lower inpatient and outpatient (primary care, urgent and nonurgent emergency department services) costs than either younger or older patients (pvalue < 0.05 and pvalue < 0.01, respectively) [39]. The second, conducted in eight Asia Pacific area countries, found extremes of age (<10 yrs and >60 yrs) to be significantly predictive of higher asthma related costs [31].

#### Gender

Gender was also found to have an association with the cost of asthma. Females were found to have higher direct costs than males, independently of the severity of disease in three high-quality studies [13,16,37].

#### Race

We also found a correlation between race and the cost of asthma. Identified Caucasian patients, particularly females in a high-quality study over one year, were found to have significantly more primary care visits (at higher costs) compared with African Americans [14]. In contrast, African-American females were found to have significantly more emergency department visits and costs compared with Caucasian patients [39].

#### Other Factors

Besides disease severity, current use of preventive drugs, current use of emergency services and/or current hospitalization was found to be the predictors of costs of childhood asthma [62]. Not surprisingly ICU admission was found to be significantly associated, with higher total hospital costs [56]. A study in Canada reported smoking and drug plans to be significant explanatory factors of higher costs from different societal, Ministry of Health, and patient perspectives. The annual cost per patient in this study varied from $1,255 in young non smokers with no drug plan and mild disease to $5,032 in older smokers with drug plans and more severe disease [15]. Statistically significant predictors of higher total costs in another study by Ungar (2001) were worse symptoms and season of participation [42]. Also, in another high quality cost of illness study, use of peak expiratory flow rate meters, low-income status, non-Caucasian race, and longer duration of asthma were found to have a significant association with increasing cost [22]. Two studies reported free access to healthcare as a significant risk factor for increased cost of asthma [30,53]. In addition, one study reported that costs increased significantly with the occurrence of asthma symptoms in the previous year as well as possible prescribed inhaled corticosteroids [54]. A cohort study in the US found the number of β-agonists and oral corticosteroid prescriptions to be significantly associated with higher costs [34]. A study in Switzerland reported use of controller therapy versus symptom relief medication and involvement of a pulmonologist in management was associated with an 80% and 30% increase in direct medical asthma costs, respectively [35]. Another Italian study reported asthma subjects, with chronic cough and sputum production to have higher costs compared to subjects without the symptoms. Moreover, the risk of having high direct medical expenditures was reported to be significantly higher for blue-collar workers as compared to workers of a higher socio economic status [16]. Finally, multiple regression analyses of the 2,052 exacerbations included in the economic analysis showed that the cost of exacerbations was significantly affected by country (P < 0.0001). Mean costs were found to be significantly higher in secondary care ($1,994) than primary care ($657, p = 0.0003) [66].

## Discussion

Economic evaluations of asthma have been performed in many countries. But there are only limited population based studies. The studies reported here are difficult to compare because of differences in study designs, definitions of costs, and different time periods. The present systematic review was aimed at identifying the evidence concerning the economic burden of asthma, as there have been no other systematic reviews in the literature that have reviewed this field and it is a highly emergent aspect of health-services management.

Asthma is associated with enormous healthcare expenditures that includes both direct costs, in the form of hospitalizations and medications, and indirect costs, in the form of loss of work which is a combination of directly missed days of work/school that occur during the exacerbation and the loss of future potential earnings associated with both morbidity and mortality.

Indirect costs in some studies constitute a major portion of health related costs, and were identified to be higher than direct costs in six studies [13,15,17,22,51,52]. However, not all of the reviewed studies have evaluated the indirect costs associated with asthmatic patients. Therefore, additional studies are necessary to provide a clearer description of these indirect costs and their contribution to the total cost of care.

In contrast, nine cost of illness studies reported that the direct costs of asthma, associated with hospitalization or medications, to be higher than the indirect costs of asthma [11,14,18,29,42,47-50]. This may possibly be explained by the costs of hospitalization and medication as the most important cost among direct costs.

The significant contribution of direct healthcare costs due to hospitalization in studies is not surprising given the inherent high costs associated with acute care versus ambulatory care [14,15,20,28,30,36,38,42,48-50,53-57,65,72]. Variations in the cost of hospitalization are likely related to the differences in the socioeconomic environment, that is, the difference in the gross national income per capita, or more specifically differences in salary costs in different countries.

The fact that the cost of asthma medications is the largest proportion of direct costs of asthma, is likely explained by the fact that there are a relatively large proportion of patients with asthma of whom only a minority are admitted to hospital. In addition the combination of frequent use, the higher cost of newer asthma drugs [11,13,15,19,22,23,27-29,35,37,43,46,47,52,56,60], the smaller proportion in hospital costs and the higher proportion in medication costs seen in some studies could also point to better control of asthma. It also likely reflects a rise in the prevalence of asthma. Furthermore, lower hospital costs can partly be explained by an overall trend towards shorter length of stay in hospital [52].

The predominance of non urgent office and outpatient costs compared to hospitalization and emergency department charges reported in five reviewed studies might be explained by the better access of patients to preventive asthma care or fewer acute exacerbations requiring hospitalization or emergency care visits [33,37,39,40,43]. Likewise, the predominance of urgent costs might be due to the poor control of the disease in countries where primary care is less well developed, leading to more urgent healthcare utilization.

Hospital type and characteristics were reported to have been associated with differences in charges for asthma hospitalizations. Teaching hospitals were found to have higher charges compared with non-teaching hospitals [44]. This is likely due to higher costs associated with teaching hospitals in terms of funding due to teaching and also the overall higher acuity and greater likelihood of more complex patients being cared for in these hospitals.

The higher costs associated with patients admitted to medical centers and regional hospitals than other patients admitted to district hospitals can be explained partly by the tendency of medical centers and regional hospitals to receive a relatively higher proportion of patients suffering from more serious illnesses than district hospitals [32].

In contrast, two studies in the US found no significant differences in costs and charges between teaching and non-teaching hospitals suggesting that, even with the responsibility of providing education, research, and care for indigent patients, teaching hospitals are competitive in their treatment of asthma [25,59]. One possible reason contributing to the differences in costs based on hospital ownership might be attributable to a hospital's levels of efficiency and also with the exception of mechanical ventilation of asthma patients; usual care of asthma patients irrespective of location is not associated with a high level of technology [32].

Not surprisingly costs of asthma care were found to increase in the presence of exacerbations and with disease of greater severity [11,13,15,17,19,21-23,27,30,31,35,36,41,46,51,53,54,58,62,66,67].

The reasons that children with asthma reported higher healthcare costs compared to non-asthma conditions could be related to: higher use of ambulatory care and medications for upper respiratory tract infections or conditions that can act synergistically with asthma (such as respiratory infections, otitis, sinusitis, etc), the use of day-care centers, experiencing more severe asthma on average and higher healthcare use in general, and misclassification of asthma-related encounters as "non-asthma" [24,25,30,40-43,57,62,70,71]. However, for adults, the increased total costs and increased costs per affected person could be explained by either an increase in the severity of asthma or a decrease in the use of effective healthcare resources. In elderly patients with comorbidities, such as congestive heart failure, which may deteriorate with asthma exacerbations, have been associated with a sharp increase in the costs derived from the disease [36,56,68].

Several studies have investigated a variety of potential risk factors associated with a higher risk of direct or indirect costs of asthma including: both old and young ages, female gender, smoking, co-morbid conditions, chronic cough and phlegm, use of peak expiratory flow rate meters, free access to healthcare, low-income status, nonwhite race, asthma symptoms in the past year, longer duration of asthma, controller therapy versus quick therapy, involvement of a pulmonologist in diagnosis or treatment, number of β-agonists and oral corticosteroid prescriptions, and whether inhaled corticosteroids had been prescribed before. The findings that females with asthma spend more on annual asthma care than males might be because of a higher use of acute care facilities due to inadequate medication and poor inhalation skills, or it might be interpreted as females confronted with a chronic illness are more concerned than males and thus, seek medical care more often and have more medicine prescribed.

Three more recent studies have been reviewed in this systematic review; however, their findings didn't contribute further to the previous results [73-75].

Despite the availability of effective preventive therapy, hospital admissions from acute asthma are increasing. This might demonstrate that patients with acute asthma exacerbations continue to be treated inappropriately prior to hospital admission.

## Conclusion

In summary, asthma is not only associated with patient specific impairment, but it is also associated with a significant cost to society. The comparison of studies assessing direct and indirect costs of asthma underscores important facts: hospitalization and medications have been found to be the most important cost driver of direct costs, while work/school absenteeism accounted for the greatest percentage of indirect costs. The cost of asthma was found to be strongly correlated with comorbidities, age, severity of disease, and some other factors. It was also found to vary significantly by hospital ownership, location, and teaching status. A large variation of asthma control can partly be explained by variation in guideline adherence to medication use and deficits of patients' management especially as it relates to access to patient education. Particular interventional strategies such as intensive education of patients and physicians, regular follow-up and preplanned homecare are required to improve quality of life as well as decrease the economic burdens of asthma.

## Abbreviations

ACT: Asthma Control Test; AIDS: Acquired immune deficiency syndrome; AUD: Australian dollar; CAD: Canadian dollar; CDSR: Cochrane Database of Systematic Reviews; CINAHL: Cumulative Index to Nursing and Allied Health Literature; ED: Emergency Department; EUR: The euro currency code; GBP: Great Britain pound; ICER: Incremental cost effectiveness ratio; ICU: Intensive Care Unit; OHE-HEED: Health Economic Evaluation Database; SD: Standard Deviation; UK: United Kingdom; USD: United States dollar

## Competing interests

The authors declare that they have no competing interests.

## Authors' contributions

KB edited and prepared the final manuscript for publication. KB and KA were responsible for reviewing articles, judging their relevance, assessing their quality, and extracting data. MW assisted in literature search. MF, MW, JS, CM, and LL critically reviewed drafts of this article. MF supervised the literature review, edited the manuscript and provided general oversight. All authors read and approved the final manuscript.

## Pre-publication history

The pre-publication history for this paper can be accessed here:


